# Risk of subsequent gliomas and meningiomas among 69,460 5-year survivors of childhood and adolescent cancer in Europe: the PanCareSurFup study

**DOI:** 10.1038/s41416-024-02577-y

**Published:** 2024-01-19

**Authors:** Emma J. Heymer, Michael M. Hawkins, David L. Winter, Jop C. Teepen, Ceren Sunguc, Cécile M. Ronckers, Rodrigue S. Allodji, Daniela Alessi, Elaine Sugden, Fabiën N. Belle, Francesca Bagnasco, Julianne Byrne, Edit Bárdi, Stanislaw Garwicz, Desiree Grabow, Momcilo Jankovic, Peter Kaatsch, Melanie Kaiser, Gisela Michel, Christina Schindera, Nadia Haddy, Neige Journy, Maja Česen Mazić, Roderick Skinner, Judith L. Kok, Maria W. Gunnes, Thomas Wiebe, Carlotta Sacerdote, Milena M. Maule, Monica Terenziani, Zsuzsanna Jakab, Jeanette F. Winther, Päivi M. Lähteenmäki, Lorna Zadravec Zaletel, Riccardo Haupt, Claudia E. Kuehni, Leontien C. Kremer, Florent de Vathaire, Lars Hjorth, Raoul C. Reulen

**Affiliations:** 1 Centre for Childhood Cancer Survivor Studies, Institute of Applied Health Research, University of Birmingham, Birmingham, UK; 2 Princess Máxima Center for Pediatric Oncology, Utrecht, The Netherlands; 3 German Childhood Cancer Registry, Division of Childhood Cancer Epidemiology, Institute of Medical Biostatistics, Epidemiology and Informatics (IMBEI), Johannes-Gutenberg University Mainz, Mainz, Germany; 4 Radiation Epidemiology Team, Center for Research in Epidemiology and Population Health, INSERM U1018, University Paris Saclay, Gustave Roussy, Villejuif, France; 5 Childhood Cancer Registry of Piedmont, Cancer Epidemiology Unit, Department of Medical Sciences, University of Turin and CPO-Piemonte, AOU Città della Salute e della Scienza di Torino, Turin, Italy; 6 Childhood Cancer Research Group, Institute of Social and Preventive Medicine, University of Bern, Bern, Switzerland; 7 Center for Primary Care and Public Health (Unisanté), University of Lausanne, Lausanne, Switzerland; 8 Scientific Directorate, IRCCS Istituto Giannina Gaslini, Genova, Italy; 9 Boyne Research Institute, c/o no. 1, The Maples, Bettystown, Co Meath, A92 C635 Ireland; 10 St Anna Children’s Hospital, Vienna, Austria; 11 Department of Paediatrics and Adolescent Medicine, Johannes Kepler University Linz, Kepler University Hospital, Linz, Austria; 12 Department of Clinical Sciences Lund, Paediatrics, Skane University Hospital, Lund University, Lund, Sweden; 13 Pediatric Clinic, University of Milano-Bicocca, Hospital San Gerardo, Via Donizetti 33, Monza, Italy; 14 Department of Health Sciences and Medicine, University of Lucerne, Lucerne, Switzerland; 15 Division of Pediatric Oncology/Haematology, University Children’s Hospital Basel, University of Basel, Basel, Switzerland; 16 University Children’s Hospital Ljubljana, University Medical Centre Ljubljana, Ljubljana, Slovenia; 17 Great North Children’s Hospital, Newcastle upon Tyne Hospitals NHS Foundation Trust, and Newcastle University Centre for Cancer, Newcastle University, Newcastle upon Tyne, UK; 18 Division of Paediatric and Adolescent Medicine, Oslo University Hospital Rikshospitalet, Oslo, Norway; 19 Department of Registration, Cancer Registry of Norway, Oslo, Norway; 20 Pediatric Oncology Unit, Fondazione IRCCS Istituto Nazionale dei Tumori, Milano, Italy; 21 Hungarian Childhood Cancer Registry, 2nd Department of Pediatrics, Semmelweis University, Budapest, Hungary; 22 Danish Cancer Society Research Center, Childhood Cancer Research Group, Copenhagen, Denmark; 23 Department of Clinical Medicine, Faculty of Health, Aarhus University and University Hospital, Aarhus, Denmark; 24 Department of Pediatrics and Adolescent Medicine, Turku University and Turku University Hospital, Turku, Finland; 25 Division of Radiotherapy, Institute of Oncology, Ljubljana, Slovenia; 26 DOPO Clinic, Division of Hematology/Oncology, IRCCS Istituto Giannina Gaslini, Genova, Italy; 27 Division of Pediatric Hematology/Oncology, Department of Paediatrics, University Children’s Hospital of Bern, University of Bern, Bern, Switzerland; 28 Emma Children’s Hospital, Amsterdam UMC, Amsterdam, The Netherlands

**Keywords:** Risk factors, Paediatric cancer, Cancer epidemiology, CNS cancer, Epidemiology

## Abstract

**Background:**

Childhood cancer survivors are at risk of subsequent gliomas and meningiomas, but the risks beyond age 40 years are uncertain. We quantified these risks in the largest ever cohort.

**Methods:**

Using data from 69,460 5-year childhood cancer survivors (diagnosed 1940–2008), across Europe, standardized incidence ratios (SIRs) and cumulative incidence were calculated.

**Results:**

In total, 279 glioma and 761 meningioma were identified. CNS tumour (SIR: 16.2, 95% CI: 13.7, 19.2) and leukaemia (SIR: 11.2, 95% CI: 8.8, 14.2) survivors were at greatest risk of glioma. The SIR for CNS tumour survivors was still 4.3-fold after age 50 (95% CI: 1.9, 9.6), and for leukaemia survivors still 10.2-fold after age 40 (95% CI: 4.9, 21.4). Following cranial radiotherapy (CRT), the cumulative incidence of a glioma in CNS tumour survivors was 2.7%, 3.7% and 5.0% by ages 40, 50 and 60, respectively, whilst for leukaemia this was 1.2% and 1.7% by ages 40 and 50. The cumulative incidence of a meningioma after CRT in CNS tumour survivors doubled from 5.9% to 12.5% between ages 40 and 60, and in leukaemia survivors increased from 5.8% to 10.2% between ages 40 and 50.

**Discussion:**

Clinicians following up survivors should be aware that the substantial risks of meningioma and glioma following CRT are sustained beyond age 40 and be vigilant for symptoms.

## Introduction

Currently, 81% of children diagnosed with cancer in Europe survive at least 5 years [1] with approximately one in every 1000 individuals in Europe now being a childhood cancer survivor [2]. However, survivors of cancer diagnosed before age 20 (i.e. childhood cancer survivors) are at increased risk of many long-term adverse health outcomes, including subsequent primary neoplasms (SPNs) [3–6]. Subsequent gliomas and meningiomas pose a serious risk accounting for substantial morbidity. Previous exposure to cranial irradiation during childhood cancer treatment is the primary risk factor [3–9], however, the long-term risk of developing a glioma or meningioma among survivors, particularly beyond age 40, is unknown. As the population of long-term survivors is growing, even a small excess risk sustained into old age could affect many survivors.

This study was conducted using the PanCare Childhood and Adolescent Cancer Survivor Care and Follow-Up Studies (PanCareSurFup) data of 69,460 5-year survivors of childhood cancer from 12 European countries [10, 11]. The principal aims of this large cohort study were to quantify: (1) the long-term risk of developing a glioma and meningioma in childhood cancer survivors, particularly beyond age 40; and (2) variations in the risk by demographic and cancer related factors. This is the largest cohort of childhood cancer survivors with follow-up beyond age 40 for 25% of individuals, including over a thousand gliomas and meningiomas—more than three times that included in any previous study [3, 4, 7, 8, 12].

## Methods

### The PanCare childhood and adolescent cancer survivor care and follow-up studies

The Pan-European Network for Care of Survivors after Childhood and Adolescent Cancer (PanCare) is a network of healthcare professionals, academic researchers, childhood cancer survivors and their families with representation from most European countries [10]. The PanCareSurFup project was set up to investigate the risks of cardiac disease, SPNs and late mortality in childhood cancer survivors [11]. The data relating to SPNs was collected from 13 cohorts across 12 countries (eAppendix Table 1) [13]. Ethics approvals were obtained for each cohort independently.

### Cohort ascertainment

For each country, morphology and topography codes relating to the childhood cancer diagnosis were converted into the third revision of the International Classification of Disease Oncology (ICD-O-3) by the IARC/IACR Cancer Registry Tools software [14]. Langerhans cell histiocytosis (*n* = 246), myelodysplastic syndromes (*n* = 95), immunoproliferative diseases (*n* = 2), chronic myeloproliferative and lymphoproliferative disorders (*n* = 188) were not ascertained by all countries and excluded. Neoplasms that were not classifiable according to the International Classification of Childhood Cancers (third edition) [15] were also excluded (*n* = 785), as were all non-malignant tumours except benign intracranial tumours (*n* = 873) [16, 17]. Ultimately, 69,460 5-year survivors of cancer diagnosed between 1940 and 2008, before age 20, were included.

### Subsequent primary neoplasm (SPN) ascertainment

Methods of CNS SPN ascertainment varied by country (eAppendix Table 1). The majority of subsequent primary gliomas were diagnosed through histological examination of tumour tissue (66.7%) (Table 1). For subsequent primary meningiomas, a similar proportion was diagnosed through histological examination of tumour tissue (48.9%) as through clinical examination (41.3%), which typically involved radiological assessment. Only CNS SPNs with a different histology to the original childhood cancer were included [11]. CNS SPNs with behaviour codes indicating benign (0), uncertain (benign/malignant) (1), in situ (2) or malignant (3) primary behaviour were included (behaviour codes 6 and 9 excluded, *n* = 278). ICD site was used to identify any CNS SPN, which were then categorised into gliomas and meningiomas by ICD-O-3 [18]. The 2007 WHO classification of CNS tumours was applied [18], with minor adaptations [19], to further classify subsequent primary gliomas into low-grade (grade I and II) and high-grade (grade III and IV) tumours.

**Table 1:** Characteristics of 69,460 5-year survivors in the PanCareSurFup study and number of subsequent primary gliomas and meningioma of the central nervous system.

Factor	Exposure	Survivors (%)	Glioma^a^	Meningioma^a^
Overall	Overall	69,460 (100%)	279 (100%)	761 (100%)
Sex	Male	37,738 (54.3%)	154 (55.2%)	355 (46.6%)
	Female	31,722 (45.7%)	125 (44.8%)	406 (53.4%)
Childhood	Leukaemia	16,646 (24.0%)	68 (24.4%)	335 (44.0%)
cancer	Hodgin lymphoma	6046 (8.7%)	10 (3.6%)	14 (1.8%)
type	Non-Hodgkin lymphoma	4078 (5.9%)	10 (3.6%)	31 (4.1%)
	CNS tumour^b^	14,592 (21.0%)	132 (47.3%)	319 (41.9%)
	Neuroblastoma	3178 (4.6%)	6 (2.2%)	2 (0.3%)
	Retinoblastoma	2590 (3.7%)	9 (3.2%)	30 (3.9%)
	Wilms tumour	4783 (6.9%)	7 (2.5%)	2 (0.3%)
	Bone sarcoma	3173 (4.6%)	7 (2.5%)	4 (0.5%)
	Soft-tissue sarcoma	4531 (6.5%)	18 (6.5%)	14 (1.8%)
	Other^c^	9843 (14.2%)	12 (4.3%)	2 (0.3%)
Age at	0–4 years	22,013 (31.7%)	112 (40.1%)	336 (44.2%)
childhood	5–9 years	17,672 (25.4%)	89 (31.9%)	242 (31.8%)
cancer	10–14 years	14,747 (21.2%)	52 (18.6%)	152 (20.0%)
	15–20 years	15,028 (21.6%)	26 (9.3%)	31 (4.1%)
Country	United Kingdom	17,960 (25.9%)	142 (50.9%)	470 (61.8%)
	France	3138 (4.5%)	27 (9.7%)	10 (1.3%)
	Hungary	4885 (7.0%)	6 (2.2%)	22 (2.9%)
	Italy	8966 (12.9%)	9 (3.2%)	23 (3.0%)
	Netherlands	6044 (8.7%)	17 (6.1%)	88 (11.6%)
	Denmark	4840 (7.0%)	10 (3.6%)	26 (3.4%)
	Sweden	7709 (11.1%)	23 (8.2%)	21 (2.8%)
	Norway	3783 (5.4%)	3 (1.1%)	3 (0.4%)
	Finland	6229 (9.0%)	27 (9.7%)	65 (8.5%)
	Iceland	275 (0.4%)	0 (0%)	3 (0.4%)
	Slovenia	1252 (1.8%)	7 (2.5%)	19 (2.5%)
	Switzerland	4379 (6.3%)	8 (2.9%)	11 (1.4%)
Era	<1970	8993 (12.9%)	51 (18.3%)	143 (13.8%)
childhood	1970–1979	13,479 (19.4%)	93 (33.3%)	314 (41.3%)
cancer	1980–1989	20,900 (30.1%)	90 (32.3%)	259 (34.0%)
diagnosis	1990–1999	19,260 (27.7%)	40 (14.3%)	44 (5.8%)
	2000–2008	6828 (9.8%)	5 (1.8%)	1 (0.1%)
Attained age	<20 years	15,405 (22.2%)	106 (38.0%)	54 (7.1%)
	20–29 years	18,877 (27.2%)	65 (23.3%)	201 (26.4%)
	30–39 years	17,144 (24.7%)	64 (22.9%)	301 (39.6%)
	40–49 years	10,970 (15.8%)	28 (10.0%)	150 (19.7%)
	50+ years	7064 (10.2%)	16 (5.7%)	55 (7.2%)
Proof of	Histology	–	186 (66.7%)	372 (48.9%)
diagnosis	Clinical examination (incl.radiology)	–	51 (18.3%)	314 (41.3%)
for SPN	Unknown	–	42 (15.1%)	75 (9.9%)

*N* number, *SPN* subsequent primary neoplasm, *CNS* central nervous system.

^a^A total of 279 glioma and 761 meningioma SPNs were observed among 941 survivors.

^b^CNS tumour category includes: astrocytomas (*n* = 6023), intracranial/intraspinal embryonal tumours (*n* = 1975), other gliomas (*n* = 1890), other specified cns tumours (*n* = 1657), ependymomas and choroid plexus tumour (*n* = 1378), unspecif. cns tumours (*n* = 1227), cns germ cell tumours (*n* = 433), unspecified (*n* = 9).

^c^“Other” category includes malignant gonadal germ cell tumours (*n* = 2300), malignant melanomas (*n* = 1458), thyroid carcinomas (*n* = 1295), other and unspecif. carcinomas (*n* = 1181), other unspecif. malignant tumours (*n* = 641), malignant extracranial/extragonadal germ cell tumours (*n* = 433), skin carcinomas (*n* = 423), unspec. Lymphomas (*n* = 402), hepatoblastoma (*n* = 319), misc. lymphoreticular neoplasms (*n* = 279), other and unspecif. malignant gonadal tumours (*n* = 221), gonadal carcinomas (*n* = 200), nasopharyngeal carcinomas (*n* = 194), renal carcinomas (*n* = 124), other peripheral nervous cell tumours (*n* = 94), adrenocortical carcinomas (*n* = 86), hepatic carcinomas (*n* = 84), other specif. malignant tumours (*n* = 54), unspecif. malignant renal tumours (*n* = 35), unspecif. malignant hepatic tumours (*n* = 15), unspecified (*n* = 5).

### General population cancer rates

Incidence rates for gliomas among the general population were required to compare the observed numbers among the survivors with the expected numbers from the general population. Expected rates were only derived for gliomas due to likely under-ascertainment of meningiomas among the general population alongside the potential for surveillance bias resulting in relative over-ascertainment among survivors. As such, only relative risks (RRs) could be estimated for meningiomas (see “Statistical analyses“). Incidence rates by ICD-O morphology were only available from the United Kingdom (England and Wales only) and Finland. Finnish rates were used for all Nordic countries based on geography and similarities in health care systems. UK rates were used for all other countries.

### Statistical analyses

Follow-up began 5 years after childhood cancer diagnosis and ended at the first occurrence of death, loss to follow-up, or study end date (eAppendix Table 1). Multiple gliomas per individual were allowed in all analyses involving observed and expected numbers. Standardized incidence ratios (SIRs) were calculated as the observed over the expected number of gliomas. Absolute excess risks (AERs) per 10,000 person-years were calculated as the observed minus the expected number of gliomas, multiplied by 10,000 and divided by person-years at risk. The AER can be interpreted as the excess number of gliomas observed beyond that expected per 10,000 person-years. The expected number of gliomas was calculated by multiplying the person-years for each sex, age (5-year categories), and calendar year (1-year categories) stratum by the corresponding glioma incidence rate amongst the general population and then summing across the strata. SIRs and AERs were stratified by the factors: cranial radiotherapy (CRT), sex, childhood cancer type, age at childhood cancer diagnosis, era of childhood cancer diagnosis, and attained age. To investigate the effect of each factor after having adjusted for potential confounders, multivariable Poisson regression models that included a random intercept for each country were used [20]. Directed acyclic graphs [21] and evidence from current literature [22] were used to guide the choice of potential set of confounders to include in each Poisson regression model. RRs derived from these Poisson regression models can be interpreted as a ratio of SIRs, having adjusted for potential confounders [23]. For analyses including the factor CRT, we assumed that survivors of CNS tumour and leukaemia treated with radiotherapy had received CRT, and all other survivors—irrespective of radiotherapy status—had not. Sensitivity analyses were conducted including only data providers with less than 30% of treatment data missing. As these results were similar, results including all data providers are presented. For meningiomas, similar multivariable Poisson regression models as for gliomas were used, but with the person-years as the log-offset. Cumulative incidence for the first occurrence of a relevant CNS SPN, with death treated as a competing risk, was calculated using the stcompet command in Stata [24–26].

Likelihood-ratio tests were used to test for heterogeneity and linear trend, with a two-sided *p* value < 0.05 considered statistically significant. All statistical analyses were conducted using Stata statistical software, version 16.

## Results

### Cohort characteristics

The cohort accrued 1,264,624 person-years of follow-up time and the median follow-up time from 5-year survival was 14.8 years (range: 0–70 years), with 25% of survivors at risk beyond age 40. Overall, 279 gliomas and 761 meningiomas (46 known malignant) were identified as SPNs, amongst 941 survivors (Table 1). In total, 132 (47%) gliomas and 319 (42%) meningiomas developed among CNS tumour survivors despite accounting for only 21% of the cohort. In total, 68 (24%) gliomas and 335 (44%) meningiomas developed among leukaemia survivors who accounted for 24% of the cohort.

### Risk of glioma

**Fig. 1:**
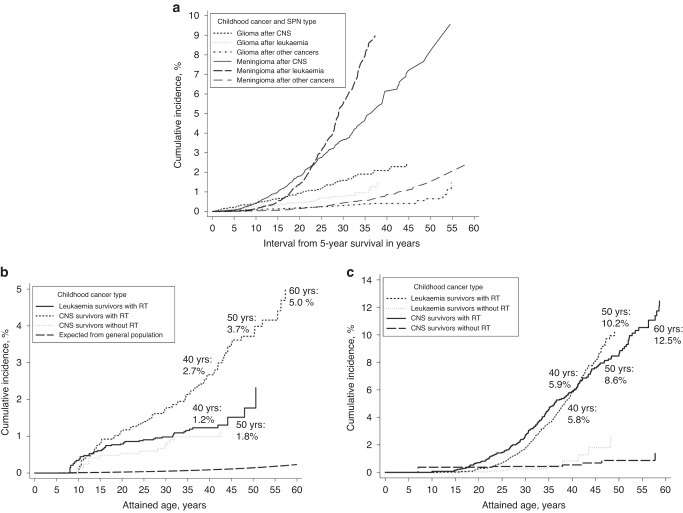
The cumulative incidence of developing (a) a glioma and meningioma by interval from 5-year survival separately among CNS, leukaemia, and other cancer survivors, (b) a glioma by attained age among CNS and leukaemia survivors stratified by whether received cranial radiotherapy treatment or not, and (c) a meningioma by attained age among CNS and leukaemia survivors stratified by whether received cranial radiotherapy treatment or not.

Overall, childhood cancer survivors were 7.5-times more likely to develop a glioma than the general population (95% CI: 6.7, 8.5) and experienced 1.9 (95% CI: 1.7, 2.2) excess gliomas per 10,000 person-years (Table 2). Survivors of each specific type of childhood cancer were at increased multiplicative (SIR) and absolute (AER) excess risk of glioma. Gliomas were most frequently observed following any CNS tumour (*n* = 132) or leukaemia (*n* = 68); together accounting for over 70% of all observed gliomas. Excess risk was highest following a CNS tumour, with 16.2-times the SIR compared to the general population (95% CI: 13.7, 19.2). Among those with known CRT status who developed a glioma, 61% had received prior CRT, however, among survivors treated for a primary CNS tumour or leukaemia and who developed a glioma, 85% and 95% had received prior CRT. CNS tumour survivors treated with CRT were at highest risk of glioma; 27-times the risk among the general population (95% CI: 21.5, 33.2) (Table 3). CNS tumour survivors treated with CRT had three times the RR of survivors treated without CRT (RR = 3.3, 95% CI: 1.8, 6.1). The SIR following CRT was particularly high for high-grade glioma (SIR = 39.0, 29.4, 51.7), although the SIR of developing low-grade glioma following CRT was still 21-fold expected (SIR = 21.0, 95% CI: 15.0, 29.4). Nonetheless, the SIR for low-grade glioma was also substantially elevated among survivors treated without CRT (SIR = 12.1, 95% CI: 6.5, 22.5). Even after adjustment for CRT, the RR of developing low-grade glioma varied with CNS tumour type (*P*_heterogeneity_ = 0.02) with survivors of a first primary meningioma at greatest risk (SIR = 43.2, 19.4, 96.1). There was no such variation in RRs by CNS tumour type in relation to high-grade gliomas (*P*_heterogeneity_ = 0.51). Both SIRs and RRs decreased with increasing attained age (*P*_trend_ ≤ 0.01), but the SIR was still 4.3-fold beyond age 50 years (95% CI: 1.9, 9.6). At 20 years of attained age almost 1% of CNS tumour survivors treated with CRT had developed a glioma, reaching 2.7% by age 40, 3.7% by age 50, and 5.0% by age 60—compared to 0.2% expected by age 60 (Fig. 1a). For CNS tumour survivors treated without CRT the cumulative incidence was 1.0% by age 40 (Fig. 1b).

**Table 2:** Standardised incidence ratios and absolute excess risks for subsequent primary gliomas by childhood cancer diagnosis.

		All glioma			Glioma grade I–II			Glioma grade III–IV	
Childhood cancer type	Obs	SIR (95% CI)	AER (95% CI)	Obs	SIR (95% CI)	AER (95% CI)	Obs	SIR (95% CI)	AER (95% CI)
Overall	279	7.5 (6.7,8.5)	1.9 (1.7,2.2)	136	6.0 (5.1,7.1)	0.9 (0.7,1.1)	143	12.0 (10.2,14.2)	1.0 (0.9,1.2)
CNS tumour	132	16.2 (13.7,19.2)	4.7 (3.9,5.6)	72	14.4 (11.4,18.2)	2.5 (2.0,3.3)	60	22.4 (17.4,28.8)	2.2 (1.7,2.8)
Leukaemia	68	11.2 (8.8,14.2)	2.4 (1.8,3.1)	23	6.5 (4.3,9.8)	0.8 (0.5,1.2)	45	25.2 (18.8,33.7)	1.7 (1.2,2.3)
Soft tissue sarcoma	18	6.1 (3.9,9.7)	1.6 (0.9,2.8)	9	5.1 (2.7,9.8)	0.8 (0.3,1.8)	9	9.1 (4.7,17.5)	0.9 (0.4,1.8)
Non-Hodgkin lymphoma	10	4.6 (2.5,8.5)	1.1 (0.5,2.5)	4	3.2 (1.2,8.6)	0.4 (0.1,1.6)	6	7.6 (3.4,17.0)	0.7 (0.3,1.9)
Retinoblastoma	9	4.5 (2.3,8.6)	1.0 (0.4,2.3)	4	3.4 (1.3,9.0)	0.4 (0.1,1.6)	5	7.1 (3.0,17.1)	0.6 (0.2,1.7)
Neuroblastoma	6	4.0 (1.8,9.0)	0.7 (0.3,2.1)	4	4.6 (1.7,12.2)	0.5 (0.1,1.8)	2	4.5 (1.1,18.2)	0.3 (0.0,1.5)
Bone Sarcoma	7	3.5 (1.7,7.4)	0.9 (0.3,2.4)	6	5.0 (2.3,11.2)	0.8 (0.3,2.3)	1	1.4 (0.2,1.0)	0.1 (0.0,41.6)
Hodgkin lymphoma	10	3.0 (1.6,5.6)	0.7 (0.3,1.7)	6	3.0 (1.4,6.8)	0.4 (0.1,1.3)	4	3.4 (1.3,9.1)	0.3 (0.1,1.1)
Wilms tumour	7	2.6 (1.2,5.4)	0.4 (0.1,1.3)	4	2.5 (0.9,6.6)	0.2 (0.0,1.1)	3	3.5 (1.1,10.7)	0.2 (0.0,1.0)
Other	12	1.9 (1.1,3.4)	0.3 (0.1,1.0)	4	1.0 (0.4,2.6)	–	8	4.6 (2.3,9.1)	0.3 (0.1,0.8)

**Table 3:** Standardised incidence ratios, absolute excess risks, and relative risks for developing a subsequent primary glioma (including by low and high-grade glioma) for central nervous system tumour survivors.

		All glioma				Low-grade glioma				High-grade glioma			
Exposure	Level	Obs	SIR (95% CI)	AER (95% CI	RR (95% CI)^a^	Obs	SIR (95% CI)	AER (95% CI	RR (95% CI)^a^	Obs	SIR (95% CI)	AER (95% CI)	RR (95% CI)^a^
Overall	All	132	16.2 (13.7,19.2)	4.7 (3.9,5.6)	–	72	14.4 (11.4,18.2)	2.5 (2.0,3.3)	–	60	22.4 (17.4,28.8)	2.2 (1.7,2.8)	–
Cranial radiotherapy^b^	No	14	8.3 (4.9,14.1)	2.3 (1.3,4.1)	1.0 (ref.)	10	12.1 (6.5,22.5)	1.7 (0.9,3.3)	1.0 (ref.)	4	5.7 (2.1,15.1)	0.6 (0.2,2.0)	1.0 (ref.)
	Yes	82	26.7 (21.5,33.2)	7.8 (6.2,9.7)	3.3 (1.8–6.1)	34	21.0 (15.0,29.4)	3.2 (2.2,4.5)	2.0 (0.9–4.3)	48	39.0 (29.4,51.7)	4.6 (3.4,6.2)	6.6 (2.3–18.8)
	Unknown	36	10.6 (7.7,14.7)	3.0 (2.1,4.3)	1.2 (0.6–2.7)	28	11.0 (7.6,15.9)	2.4 (1.6,3.5)	1.0 (0.4–2.6)	8	10.8 (5.4,21.6)	0.7 (0.3,1.4)	1.5 (0.5–5.1)
	*P*-hetero		<0.001	<0.001	<0.001		0.03	0.20	0.13		<0.001	<0.001	<0.001
1st primary CNS tumour^c^	Astrocytoma	62	14.4 (11.2,18.4)	4.1 (3.2,5.4)	1.0 (ref.)	39	14.7 (10.7,20.1)	2.6 (1.9,3.6)	1.0 (ref.)	23	16.2 (10.8,24.4)	1.5 (1.0,2.4)	1.0 (ref.)
	Ependymoma	11	15.8 (8.7,28.5)	4.5 (2.4,8.4)	0.8 (0.4–1.6)	5	11.9 (5.0,28.7)	2.0 (0.8,5.2)	0.6 (0.2–1.6)	6	25.8 (11.6,57.4)	2.5 (1.1,5.8)	1.1 (0.5–2.8)
	Medulloblastoma	22	26.3 (17.3,39.9)	6.9 (4.5,10.6)	0.7 (0.4–1.3)	9	18.7 (9.8,36.0)	2.8 (1.4,5.5)	0.5 (0.2–1.2)	13	45.8 (26.6,78.9)	4.1 (2.4,7.2)	1.1 (0.5–2.2)
	Other malignant	15	13.2 (7.9,21.8)	4.0 (2.3,7.0)	1.0 (0.6–1.8)	7	9.9 (4.7,20.7)	1.8 (0.8,4.2)	0.8 (0.4–1.9)	8	21.4 (10.7,42.8)	2.2 (1.1,4.6)	1.3 (0.6–2.9)
	Other benign	16	17.0 (10.4,27.8)	5.1 (3.0,8.5)	1.3 (0.8–2.3)	6	10.1 (4.6,22.6)	1.8 (0.7,4.4)	0.8 (0.4–2.0)	10	32.8 (17.6,60.9)	3.3 (1.7,6.2)	2.0 (0.9–4.2)
	Meningioma	6	28.7 (12.9,63.8)	9.6 (4.2,22.0)	3.8 (1.6–8.9)	6	43.2 (19.4,96.1)	9.7 (4.3,22.1)	5.5 (2.2–13.4)	0	–	–	–
	*P*-hetero		0.17	0.29	0.09		0.16	0.14	0.02		0.03	0.05	0.51
Sex^d^	Male	71	14.5 (11.5,18.2)	4.7 (3.7,6.0)	1.0 (ref.)	42	13.7 (10.1,18.7)	2.6 (1.9,3.7)	1.0 (ref.)	30	17.6 (12.3,25.1)	2.0 (1.4,2.9)	1.0 (1.0–1.0)
	Female	61	18.9 (14.7,24.3)	4.7 (3.6,6.1)	1.4 (1.0–1.9)	32	15.4 (10.9,21.8)	2.4 (1.7,3.5)	1.1 (0.7–1.8)	30	30.9 (21.6,44.2)	2.4 (1.6,3.4)	1.8 (1.1–3.0)
	*P*-hetero		0.13	0.99	0.07		0.62	0.76	0.59		0.02	0.55	0.03
Era of childhood cancer^e^	<1970	24	7.1 (4.8,10.6)	2.5 (1.6,4.0)	1.0 (ref.)	10	4.8 (2.6,9.0)	1.0 (0.4,2.1)	1.0 (ref.)	14	11.3 (6.7,19.1)	1.6 (0.9,2.8)	1.0 (ref.)
	1970–1979	43	19.9 (14.7,26.8)	5.8 (4.2,7.9)	1.9 (1.1–3.3)	21	15.2 (9.9,23.3)	2.8 (1.8,4.4)	2.0 (0.9–4.4)	22	32.0 (21.1,48.6)	3.0 (2.0,4.6)	1.7 (0.8–3.5)
	1980–1989	41	22.1 (16.3,3)	5.3 (3.9,7.3)	2.1 (1.2–3.6)	26	22.8 (15.5,33.5)	3.4 (2.3,5.0)	3.0 (1.4–6.5)	15	27.5 (16.6,45.6)	2.0 (1.2,3.3)	1.0 (0.4–2.3)
	1990–2008	24	32.4 (21.7,48.3)	6.2 (4.1,9.4)	3.7 (1.8–7.3)	15	37.8 (22.8,62.7)	3.9 (2.3,6.6)	6.3 (2.5–15.8)	9	43.8 (22.8,84.3)	2.3 (1.2,4.6)	1.2 (0.4–3.6)
	*P*-trend		<0.001	0.01	<0.001		<0.001	<0.001	<0.001		<0.001	0.31	0.99
Age at childhood cancer^f^	0–4 years	40	31.2 (22.9,42.6)	7.2 (5.3,1)	1.0 (ref.)	27	33.5 (23.0,48.8)	4.9 (3.3,7.2)	1.0 (ref.)	13	37.6 (21.8,64.7)	2.4 (1.4,4.1)	1.0 (ref.)
	5–9 years	53	21.7 (16.6,28.4)	5.7 (4.3,7.6)	0.8 (0.5–1.3)	27	18.6 (12.8,27.2)	2.9 (1.9,4.3)	0.7 (0.4–1.2)	26	31.8 (21.6,46.7)	2.8 (1.9,4.2)	1.1 (0.6–2.2)
	10–14 years	28	10.3 (7.1,14.9)	3.2 (2.1,4.8)	0.5 (0.3–0.9)	11	7.0 (3.9,12.7)	1.2 (0.6,2.4)	0.4 (0.2–0.8)	17	16.6 (10.3,26.7)	2.0 (1.2,3.3)	0.8 (0.4–1.6)
	15–20 years	11	6.5 (3.6,11.7)	2.2 (1.1,4.4)	0.4 (0.2–0.9)	7	6.0 (2.8,12.5)	1.4 (0.6,3.3)	0.4 (0.2–1.1)	4	8.2 (3.1,21.7)	0.8 (0.3,2.5)	0.5 (0.1–1.6)
	*P*-trend		<0.001	<0.001	<0.001		<0.001	<0.001	0.01		<0.001	0.11	0.17
Attained age^g^	<20 years	49	39.4 (29.8,52.2)	6.2 (4.7,8.3)	1.0 (ref.)	36	40.4 (29.1,56.0)	4.6 (3.3,6.4)	1.0 (ref.)	14	83.9 (49.7,141.7)	1.8 (1.1,3.1)	1.0 (ref.)
	20–29 years	29	15.3 (10.6,22.0)	3.1 (2.1,4.6)	0.5 (0.3–0.8)	16	11.3 (6.9,18.4)	1.7 (1.0,2.8)	0.4 (0.2–0.8)	13	36.9 (21.4,63.5)	1.4 (0.8,2.5)	0.5 (0.2–1.1)
	30–39 years	30	14.3 (1,20.4)	5.0 (3.4,7.3)	0.5 (0.3–0.9)	14	9.8 (5.8,16.5)	2.2 (1.2,4.0)	0.5 (0.2–1.0)	15	26.0 (15.7,43.1)	2.6 (1.5,4.3)	0.3 (0.1–0.7)
	40–49 years	18	11.9 (7.5,18.9)	5.7 (3.4,9.4)	0.5 (0.3–1.0)	6	7.4 (3.3,16.5)	1.8 (0.7,4.5)	0.5 (0.2–1.5)	12	18.2 (10.3,32.0)	3.9 (2.2,7.1)	0.2 (0.1–0.5)
	50+ years	6	4.3 (1.9,9.6)	3.3 (1.2,9.4)	0.3 (0.1–0.8)	0	–	–	–	6	6.5 (2.9,14.5)	3.7 (1.4,9.5)	0.1 (0.0–0.3)
	*P*-trend		<0.001	<0.001	0.02		<0.001	<0.001	0.01		<0.001	0.13	<0.001

^a^For each exposure factor a separate multivariable Poisson regression model was employed with a different set of confounders included. Directed acyclic graphs (DAGs) were used to guide the choice of potential set of confounders to include in each Poisson regression model (see: https://dagitty.net/mFq5Jac). The factor “country” was incorporated in each Poisson regression model as a random effect.

^b^Adjusted for: 1st primary CNS group, age at diagnosis, attained age, era of childhood cancer, sex.

^c^Adjusted for: sex, era of childhood cancer, attained age, age at diagnosis, cranial radiotherapy.

^d^Adjusted for: no adjustments.

^e^Adjusted for: 1st primary CNS group, sex, attained age.

^f^Adjusted for: 1st primary CNS group, attained age.

^g^Adjusted for: era of childhood cancer, age at diagnosis.

After CNS tumour survivors, survivors of leukaemia exhibited the second-highest SIR of developing a subsequent glioma; 11.2-times that expected (95% CI: 8.8, 14.2) corresponding to 2.4 excess gliomas per 10,000 person-years (Table 4). The SIR was greatest among those treated with CRT with an SIR of 14.1 (95% CI: 10.9, 18.3), particularly high-grade gliomas (SIR = 29.5, 95% CI: 21.4, 40.7), but also low-grade gliomas (SIR = 8.7, 95% CI: 5.6, 13.4). Whilst the RR of developing a glioma decreased with attained age (*P*_trend_ = 0.03), the SIR remained high with a 10.2-fold SIR beyond age 40 years (95% CI: 4.9, 21.4). For leukaemia survivors diagnosed most recently (1990–2008) the RR of developing a glioma was 60% lower than for survivors diagnosed before 1980 (RR = 0.4, 95% CI: 0.2, 0.9). This decrease in risk by era of diagnosis was also supported by the cumulative incidence (eAppendix Fig. 1). Following CRT, the cumulative incidence of a glioma reached 1.2% by age 40 years and 1.8% by 50 years, compared to 0.1% expected (Fig. 1b).

**Table 4:** Standardised incidence ratios, absolute excess risks, and relative risks for developing a subsequent primary glioma, including low and high-grade glioma, for leukaemia survivors.

		All glioma				Low-grade glioma				High-grade glioma			
Exposure	Level	Obs	SIR (95% CI)	AER (95% CI	RR (95% CI)^a^	Obs	SIR (95% CI)	AER (95% CI	RR (95% CI)^a^	Obs	SIR (95% CI)	AER (95% CI)	RR (95% CI)^a^
Overall	All	68	11.2 (8.8,14.2)	2.4 (1.8,3.1)	–	23	6.5 (4.3,9.8)	0.8 (0.5,1.2)	–	45	25.2 (18.8,33.7)	1.7 (1.2,2.3)	–
Cranial radiotherapy^b^	No	3	2.7 (0.9,8.4)	0.4 (0.1,2.3)	1.0 (ref.)	1	1.7 (0.2,12.1)	0.1 (9.5)	1.0 (ref.)	2	6.7 (1.7,26.6)	0.3 (0.1,1.8)	1.0 (ref.)
	Yes	57	14.1 (10.9,18.3)	3.2 (2.4,4.2)	6.2 (1.9–19.9)	20	8.7 (5.6,13.4)	1.1 (0.6,1.7)	8.6 (1.0–71.9)	37	29.5 (21.4,40.7)	2.1 (1.5,3.0)	5.7 (1.3–23.8)
	Unknown	8	8.6 (4.3,17.3)	1.7 (0.8,3.7)	3.7 (1.0–14.0)	2	3.1 (0.8,12.4)	0.3 (2.5)	2.2 (0.2–27.7)	6	25.7 (11.5,57.1)	1.4 (0.6,3.1)	3.4 (0.7–17.1)
	*P*-hetero		<0.001	<0.001	<0.001		0.05	0.04	0.03		0.04	0.01	<0.001
Sex^c^	Male	39	11.1 (8.1,15.2)	2.6 (1.9,3.7)	1.0 (ref.)	12	6.0 (3.4,10.6)	0.7 (0.4,1.5)	1.0 (ref.)	27	24.7 (16.9,36.0)	1.9 (1.3,2.9)	1.0 (ref.)
	Female	29	11.4 (7.9,16.3)	2.1 (1.4,3.1)	1.0 (0.6–1.7)	11	7.1 (3.9,12.8)	0.8 (0.4,1.5)	1.2 (0.5–2.7)	18	25.9 (16.3,41.1)	1.4 (0.9,2.2)	1.0 (0.6–1.9)
	*P*-hetero		0.93	0.4	0.93		0.69	0.98	0.69		0.88	0.29	0.88
Era of childhood cancer^d^	<1980	32	13.7 (9.7,19.4)	3.5 (2.4,5.1)	1.0 (ref.)	13	9.3 (5.4,15.9)	1.4 (0.7,2.5)	1.0 (ref.)	19	24.3 (15.5,38.2)	2.1 (1.3,3.4)	1.0 (ref.)
	1980–1989	26	10.2 (7.0,15.0)	2.1 (1.4,3.2)	0.6 (0.4–1.1)	6	4.0 (1.8,8.8)	0.4 (0.1,1.2)	0.3 (0.1–0.9)	20	27.9 (18.0,43.2)	1.7 (1.1,2.7)	0.6 (0.3–1.3)
	1990–2008	10	8.4 (4.5,15.6)	1.4 (0.7,2.9)	0.4 (0.2–0.9)	4	6.5 (2.4,17.2)	0.6 (0.2,1.8)	0.4 (0.1–1.3)	6	20.7 (9.3,46.1)	0.9 (0.4,2.2)	0.3 (0.1–0.9)
	*P*-trend		0.13	0.01	0.01		0.25	0.07	0.06		0.89	0.09	0.03
Age at childhood cancer^e^	0–4 years	37	16.0 (11.6,22.1)	3.2 (2.3,4.5)	1.0 (ref.)	13	9.5 (5.5,16.4)	1.1 (0.6,2.0)	1.0 (ref.)	24	40.3 (27.0,60.1)	2.2 (1.4,3.3)	1.0 (ref.)
	5–9 years	21	9.4 (6.1,14.4)	1.9 (1.2,3.1)	0.6 (0.4–1.1)	6	4.6 (2.1,10.2)	0.5 (0.2,1.3)	0.6 (0.2–1.5)	15	22.7 (13.7,37.6)	1.5 (0.9,2.5)	0.7 (0.3–1.3)
	10–20 years	10	9.3 (5.0,17.2)	2.2 (1.1,4.5)	0.7 (0.3–1.4)	4	6.6 (2.5,17.5)	0.9 (0.3,2.7)	1.1 (0.3–3.6)	6	15.7 (7.0,34.9)	1.4 (0.6,3.3)	0.6 (0.2–1.5)
	*P*-trend		<0.001	0.02	0.01		0.07	0.17	0.39		<0.001	0.05	0.03
Attained age^f^	<20 years	32	15.9 (11.2,22.4)	2.6 (1.8,3.7)	1.0 (ref.)	16	12.2 (7.4,19.8)	1.3 (0.7,2.1)	1.0 (ref.)	14	97.0 (57.4,163.7)	1.8 (1.1,3.1)	1.0 (ref.)
	20–29 years	17	9.2 (5.7,14.8)	1.8 (1.0,3.0)	0.6 (0.3–1.1)	3	2.5 (0.8,7.9)	0.2 (0.0,1.4)	0.2 (0.1–0.7)	12	39.5 (22.5,69.6)	1.3 (0.7,2.4)	0.5 (0.2–1.0)
	30–39 years	12	7.9 (4.5,14.0)	2.4 (1.3,4.7)	0.5 (0.2–1.0)	2	2.5 (0.6,1.0)	0.3 (0.0,2.8)	0.2 (0.0–0.8)	14	28.8 (17.1,48.7)	2.4 (1.4,4.1)	0.2 (0.1–0.6)
	40+	7	10.2 (4.9,21.4)	4.7 (2.1,10.7)	0.6 (0.2–1.4)	2	8.0 (2.0,31.9)	1.3 (0.3,6.4)	0.4 (0.1–2.1)	12	19.9 (11.3,35.0)	3.9 (2.2,7.1)	0.2 (0.0–0.6)
	*P*-trend		0.03	0.89	0.04		0.01	0.03	<0.001		<0.001	0.05	<0.001

^a^For each exposure factor a separate multivariable Poisson regression model was employed with a different set of confounders included. A directed acyclic graph (DAG) was used to guide the choice of potential set of confounders to include in each Poisson regression model (see: dagitty.net/mrsMx0N). The factor ‘country’ was incorporated in each Poisson regression model as a random effect.

^b^Adjusted for: age at diagnosis, attained age.

^c^Adjusted for: no adjustments.

^d^Adjusted for: attained age.

^e^Adjusted for: attained age.

^f^Adjusted for: era of childhood cancer, age at diagnosis.

### Risk of meningioma

Most meningiomas were observed in CNS tumour (*n* = 319) or leukaemia (*n* = 335) survivors (Table 1); combined these accounted for over 80% of all observed meningiomas. In all, the majority of meningioma cases (64.7%) had a history of CRT.

Among CNS tumour survivors, the RR following CRT was 13-times that without CRT (RR = 13.0, 95% CI: 6.7, 25.4). The RR was higher for those diagnosed at a younger age (*P*_trend_ < 0.001) with the RR of those diagnosed aged 15–20 half that of those diagnosed aged 0–4 years (RR = 0.5, 95% CI: 0.4, 0.7) (Table 5). The RR also consistently increased for more recent era of diagnosis (*P*_heterogeneity_ < 0.001). The RR was almost two times higher for those treated between 1990 and 2008 compared to those treated before 1970 (RR: 1.9, 95% CI: 1.2, 3.0, *P*_trend_ < 0.001). The RR increased with attained age (*P*_trend_ < 0.001); those over 40 years of age had 10-fold the risk of those under 20 (RR: 10.2, 95% CI: 6.3, 16.5).

**Table 5:** Multivariable relative risks for subsequent primary meningiomas for survivors of CNS tumours and leukaemia.

Factor	Exposure	CNS tumour	Leukaemia
		RR (95% CI)^a^	RR (95% CI)^a^
Cranial radiotherapy^b^	No	1.0 (ref.)	1.0 (ref.)
	Yes	13.0 (6.7–25.4)	5.4 (2.9–9.9)
	Unknown	6.7 (3.2–14.2)	3.0 (1.5–6.1)
	*P*_heterogeneity_	<0.001	<0.001
Sex^c^	Males	1.0 (ref.)	1.0 (ref.)
	Females	1.2 (1.0–1.5)	1.4 (1.1–1.7)
	*P*_heterogeneity_	0.13	0.01
Era of childhood cancer diagnosis^d^	<1970	1.0 (ref.)	1.0 (ref.)
	1970–1979	1.3 (1.0–1.7)	9.2 (2.3–37.2)
	1980–1989	1.6 (1.2–2.2)	7.3 (1.8–29.8)
	1990–2008	1.9 (1.2–3.0)	2.1 (0.5–9.7)
	*P*_heterogeneity_	<0.001	0.001
Age at childhood cancer diagnosis^e^	0–4 years	1.0 (ref.)	1.0 (ref.)
	5–9 years	0.8 (0.6–1.1)	0.9 (0.7–1.2)
	10–14 years	0.6 (0.5–0.9)	0.5 (0.3–0.7)
	15–20 years	0.5 (0.4–0.7)	0.2 (0.1–0.3)
	*P*_trend_	<0.001	<0.001
Attained age^f^	<20 years	1.0 (ref.)	1.0 (ref.)
	20–29 years	3.2 (2.1–4.9)	7.6 (4.6–12.6)
	30–39 years	6.9 (4.4–10.8)	22.3 (13.4–37.1)
	40+ years	10.2 (6.3–16.5)	33.6 (18.9–59.8)
	*P*_trend_	<0.001	<0.001

*CNS* central nervous system, *RR* relative risk, *CI* confidence interval.

^a^For each exposure factor a separate multivariable Poisson regression model was employed with a different set of confounders included. A directed acyclic graph (DAG) was used to guide the choice of potential set of confounders to include in each Poisson regression model (see: dagitty.net/mrsMx0N). The factor ‘country’ was incorporated in each Poisson regression model as a random effect.

^b^Adjusted for: age at diagnosis, attained age.

^c^Adjusted for: no adjustments.

^d^Adjusted for: attained age.

^e^Adjusted for: attained age.

^f^Adjusted for: era of childhood cancer, age at diagnosis.

Following CRT, leukaemia survivors were at 5-fold increased risk compared to those treated without CRT (RR: 5.4, 95% CI: 2.9, 9.9). The RR for leukaemia survivors was higher for those diagnosed at a younger age, declining by 80% for those diagnosed aged 15–20 compared to those aged 0–4 (RR = 0.2, 95% CI: 0.1, 0.3) (*P*_trend_ < 0.001) (Table 5). The RR was highest in patients diagnosed in the 1970s and 1980s (*P*_heterogeneity_ < 0.001) and increased with attained age (*P*_trend_ < 0.001); those aged over 40 had 34-fold the risk of those aged under 20 (RR = 33.6, 95% CI: 18.9, 59.8).

Among survivors treated with CRT, CNS tumour survivors had the highest cumulative incidence of meningioma, up to 40 years of age (Fig. 1c); however, beyond age 40, it was higher for leukaemia survivors. Both cumulative incidence curves increased steeply with increasing age: among CNS tumour survivors following CRT it doubled from attained age 40 to attained age 60 years from 5.9% to 12.5%; among leukaemia survivors following CRT it reached 5.8% by attained age 40 years and 10.2% by attained age 50 years (Fig. 1c). Corresponding age-specific cumulative incidence for CNS tumour survivors treated without RT were 0.5%, 0.9%, and 1.4% by age 40, 50, and 60, respectively. For leukaemia survivors initially treated without RT, they were 0.8% and 2.6% by age 40 and 50, respectively.

## Discussion

### Main findings

In this largest ever cohort study of 69,460 survivors of childhood cancer with over three times the number of CNS SPNs of any previous study [3, 4, 12], we estimated the long-term risks of gliomas and meningiomas with greater statistical power than previously possible, even into the 6th decade of life. We demonstrated that leukaemia and CNS tumour survivors remain at high risk even beyond age 40. For leukaemia survivors the cumulative incidence of meningioma doubles from age 40–50. For CNS tumour survivors the cumulative incidence of developing both glioma and meningioma doubles from age 40–60.

### Risk of glioma

Previous evidence suggests that the SIR of developing a glioma decreases with time since 5-year survival and attained age [3, 7, 8], but it is uncertain whether the SIR remains elevated beyond age 40 years with one study from the North American CCSS suggesting there is no radiation-induced risk beyond age 25 years [8]. Although SIRs also decreased with increasing attained age in the current study, the SIR for glioma was still over ten-fold beyond age 40 for both CNS and leukaemia survivors. As most survivors in the CCSS study have not reached ages beyond age 40 and 50 yet, it could be that the risks of glioma in the CCSS cohort have remained undetected but may emerge once more survivors reach ages beyond age 40. This elevated SIR sustained into older age implies that more survivors than previously suggested may be at long-term risk of developing a glioma.

Results from the CCSS study also suggested that after adjustment for cumulative radiation doses received to the brain, the risk of glioma is no longer increased among any survivors [8]. However, here we report high SIRs of glioma even among survivors not treated with CRT, particularly low-grade gliomas (SIR = 12.1, 95% CI: 6.5, 22.5). Although it could be that such survivors still received radiation to the head and neck or scatter from a tumour irradiated in lower body areas, the possibility of a genetic predisposition should not be ignored. For example, we found very high RRs of low-grade gliomas following childhood meningioma (SIR = 43.2, 95% CI: 19.4, 96.1), which may suggest that a diagnosis of NF-2 might be implicated in increasing both the risk of childhood meningioma and subsequent glioma. Other cancer syndromes such as Li-Fraumeni or NF-1 have also been associated with increased risks of developing glioma [27]. Nonetheless, the overall number of gliomas in CNS tumour survivors treated without CRT was 14 and almost all low-grade (*n* = 10), suggesting that the number of gliomas attributable to a potential genetic predisposition is likely small.

Leukaemia survivors treated in the 1970s and 1980s had higher cumulative incidence and RR of gliomas than those treated more recently, likely due to prophylactic CRT use during these decades [28]. Nonetheless, the SIR was still 8-fold for those diagnosed beyond 1990 suggesting that other treatment modalities such as total body irradiation or specific chemotherapeutic agents may also be implicated in glioma development, although strong evidence for such risk factors is currently lacking [29].

### Risk of meningioma

Most previous large-scale studies reported risks of developing meningioma up to age 40 [8, 12] or 45 [30] and even those had very few meningiomas beyond age 40. The North-American CCSS [12] reported 40 meningiomas beyond age 40 years compared to 205 here, allowing for new accurate long-term risk estimates. In the CCSS, the cumulative risk of developing meningioma following CRT was 5.6% (95% CI: 4.7, 6.7) by age 40; remarkably similar to our estimates. Similarly, in a Dutch study the cumulative incidence following CRT was 7.3% (95% CI: 4.5, 10.8) by age 45. However, our study shows for the first time that these risks following CRT continue to steeply rise with more than 12.5% of CNS tumour survivors developing a meningioma by age 60 and 10.2% of leukaemia survivors by age 50.

This study found that beyond 40 years of attained age, survivors of leukaemia treated with CRT had a higher cumulative incidence of developing meningioma than CNS tumour survivors treated with CRT. It has been postulated that leukaemia survivors may be at higher risk due to typically a larger volume of the meninges having been irradiated when being given prophylactic cranial irradiation [30]. However, a more likely explanation relates to competing risk of death. As CNS tumour survivors generally have substantially higher late mortality [31–33], the extent to which death acts as a competing risk is greater among CNS tumour survivors than leukaemia survivors. As such, the extent to which mortality prevents survivors from developing meningiomas is higher among CNS tumour survivors.

For CNS tumour survivors the excess risk steadily increased with more recent treatment era. However, for leukaemia survivors, era and excess risk seemed to relate differently, with the highest risk among those treated in the 1970s and 1980s, when CNS radiotherapy prophylaxis was in most widespread use. Before 1970, CNS prophylaxis was still being adopted, and after 1990 the move towards intrathecal methotrexate had begun.

The risk of developing a meningioma also appeared higher for those diagnosed with their first cancer at a younger age, but this is likely explained by most medulloblastoma and leukaemia survivors treated with CRT having been diagnosed at a young age.

### Clinical implications

A key gap in existing research, as identified in the recently published guidelines for CNS tumour surveillance, is the lifetime risk of developing CNS SPNs, particularly beyond 30 years after treatment [29]. Here, we were able to estimate the risks up to the 6th decade of life. We determined that cumulative incidence is substantial, with excess risks for meningioma increasing steeply with age. International Guideline Harmonization Group (IGHG) do not recommend brain MRI for surveillance of asymptomatic meningiomas after childhood cancer as there is insufficient evidence of reducing mortality and morbidity and it may even lead to overdiagnosis resulting in overtreatment [29, 34]. However, they do suggest offering an annual neurological exam to survivors treated with cranial radiotherapy. Our findings that CNS tumour and leukaemia survivors treated with CRT remain at high risks into old age emphasise the importance of offering annual neurological exams and remaining vigilant for symptoms, even for survivors over age 40 years.

### Potential limitations

A potential limitation is that cumulative doses of radiotherapy and chemotherapy along with specific genetic factors were not available on a whole cohort basis. As it would practically not be feasible to collect this information on nearly 70,000 survivors, only a large case-control study can address these long-term risks. Some previous studies have investigated risks by detailed treatment information, but with few cases compared to this study [7, 8, 35].

We assumed that CNS and leukaemia survivors treated with radiotherapy received CRT, and all other survivors—irrespective of radiotherapy status—did not. It is possible that, a few other survivors may have received CRT for CNS disease/metastases, and some leukaemia survivors may have received only non-cranial RT. Some RT information is missing, but as this is largely accounted for by cohorts from entire countries (Nordic Countries and Italian population-based cohorts) which provided no or less than 30% RT information, bias is unlikely to be substantial. Exclusion of the Nordic countries and Italian based cohort did not change our results appreciably.

Under-ascertainment of meningiomas among the general population has prevented comparison to general population meningioma rates in this study. Under-ascertainment of meningiomas among survivors may also be considered a limitation here as other studies have detected asymptomatic meningiomas in around one-fifth of leukaemia survivors treated with CRT [36, 37]. However, unidentified asymptomatic meningiomas are often less problematic to the patient than premature detection as immediate interventions may be unnecessary, but early detection can negatively impact quality of life.

## Conclusion

In conclusion, this study shows, for the first time, that substantially increased risks of meningioma and glioma are sustained beyond age 40, implying that gliomas and meningiomas following CRT will be an increasing problem in ageing survivors. One in 20 CNS tumour survivors treated with CRT had developed a glioma by age 60. Furthermore, 1 in 10 leukaemia survivors and 1 in 11 CNS tumour survivors treated with CRT had developed a meningioma by age 50. Clinicians responsible for follow-up care of CNS tumour and leukaemia survivors should be aware that the risk of gliomas and meningiomas following CRT is sustained into at least the 6th decade of life and should be vigilant in checking for symptoms. Annual neurological exams may be recommended for survivors treated with CRT.

### Supplementary information

eAppendix

## Data Availability

Access to anonymised data may be granted under conditions agreed with the relevant (local) legal and research ethics committees and with appropriate data sharing agreements and permissions from each data provider in place. Any data sharing would have to comply with the EU General Data Protection Regulation. The data that support the findings of this study are not publicly available due to privacy and ethical restrictions. Aggregated data in the form of tables may be available on reasonable request.
