# Supplementary material for: Risk of subsequent gliomas and meningiomas among 69,460 5-year survivors of childhood and adolescent cancer in Europe: the PanCareSurFup study
Source: Br J Cancer. 2024 Jan 19;130(6):976–86. doi: 10.1038/s41416-024-02577-y (PMC10951281; doi:10.1038/s41416-024-02577-y)
Supplement: Supplementary file 1 — eAppendix [file 41416_2024_2577_MOESM1_ESM.docx]

**Supplementary Information - eAppendix**

eAppendix table 1: Cohort characteristics of each of the 13 individual cohorts of partners contributing data on subsequent primary neoplasms (SPNs) to the pooled PanCareSurFup cohort study

eAppendix table 2: Childhood cancer type and radiotherapy treatment status of all 69,460 five-year survivors in the PanCareSurFup study

eAppendix table 3: Age-specific number of individuals at risk of developing a glioma or meningioma SPN

eAppendix figure 1: Cumulative incidence of gliomas following childhood leukaemia by era of diagnosis

eAppendix figure 2: Cumulative incidence of gliomas by childhood cancer type

eAppendix figure 3: Cumulative incidence of meningiomas by childhood cancer type

| **eTable 1**. Cohort characteristics of each of the 13 individual cohorts of partners contributing data on subsequent primary neoplasms (SPNs) to the pooled PanCareSurFup cohort study | | | | | | | | | |
| --- | --- | --- | --- | --- | --- | --- | --- | --- | --- |
| Cohort | No. of 5-year survivors | Period of  Diagnosis | Study design | Childhood Cancer Inclusion Criteria | Age at childhood  cancer diagnosis | Study  Exit date^\|\|\|^ | Mean follow-up time (years) | % Lost to  follow-Up | Method of SPN Ascertainment |
| France | 3,138 | 1946–1986 | Hospital–based | Malignant or intracranial, excl. Leukaemia | 0-18 yrs | Sep-14 | 26.48 | *—* | Multiple methods^$^ |
| Hungary | 4,885 | 1971–2008 | Population–based | Malignant or intracranial | 0-19 yrs | Dec-14 | 10.26 | *<5%* | Multiple methods^†^ |
| Italy (PB) | 7,476 | 1964–2005 | Population–based | Malignant or intracranial | 0-19 yrs | May-10 | 9.47 | 2% | Multiple methods^‡^ |
| Italy (HB) | 1,490 | 1960–2008 | Hospital–based | Malignant or intracranial | 0-19 yrs | Dec-12 | 15.72 | *—* | Multiple methods^‡^ |
| Netherlands | 6,044 | 1963–2001 | Population–based | Malignant or intracranial | 0-17 yrs | Dec-12 | 17.09 | 6% | Multiple methods^§^ |
| Denmark | 4,840 | 1943–1998 | Population–based | Malignant or intracranial | 0-19 yrs | Dec-03 | 16.31 | 5% | Population based* |
| Sweden | 7,709 | 1958–1998 | Population–based | Malignant or intracranial | 0-19 yrs | Dec-03 | 14.97 | 2% | Population based* |
| Norway | 3,783 | 1953–1997 | Population–based | Malignant or intracranial | 0-19 yrs | Dec-02 | 14.03 | 3% | Population based* |
| Finland | 6,229 | 1953–2006 | Population–based | Malignant or intracranial | 0-19 yrs | Dec-11 | 16.85 | 5% | Population based* |
| Iceland | 275 | 1955–1998 | Population–based | Malignant or intracranial | 0-19 yrs | Dec-03 | 12.59 | 5% | Population based* |
| Slovenia | 1,252 | 1960–2002 | Population–based | Malignant or intracranial | 0-16 yrs | Jul-14 | 19.83 | 5% | Population based* |
| Switzerland | 4,379 | 1964–2005 | Population–based | Malignant or intracranial | 0-19 yrs | Dec-13 | 10.55 | *<5%* | Multiple methods^\|\|^ |
| UK | 17,960 | 1940–1991 | Population–based | Malignant or intracranial | 0-14 yrs | Sep-16 | 28.23 | 5% | Population based* |
| Total | 69,460 | 1940-2008 |  |  | 0-19 yrs |  |  |  |  |
| Abbreviations: SPN; Subsequent primary neoplasm, PB; population-based, HB; hospital-based, UK; United Kingdom, RT; radiotherapy treatment  ^$^Multiple methods include: long-term follow-up clinics, questionnaires to survivors, national mortality records and health insurance registries.  ^†^Multiple methods include: long-term follow-up clinics, questionnaires to survivors and medical records/hospital data.  ^‡^Multiple methods include: medical records/ hospital data, national mortality records.  ^§^Multiple methods include: population-based cancer registries, long-term follow-up clinics and medical records/hospital data.  ^\|\|^Multiple methods include: population-based cancer registries, questionnaires to survivors, long-term follow-up clinics, and national mortality records.  *Population based refers to national population-based cancer registries.  ^\|\|\|^For France, Hungary, Italy-HB and Switzerland this is not the definitive study end date, but the last known date of exit, as individuals were on active follow-up so there was not one fixed study end date  — Could not calculate as there was no definitive study end date. | | | | | | | | | |

| **eTable 2.** Childhood cancer type and radiotherapy treatment status of all 69,460 five-year survivors in this study. | | | | | |
| --- | --- | --- | --- | --- | --- |
|  | | **Radiotherapy treatment^a, b^** | | | **Total** |
|  |  | **No** | **Yes** | **Unknown** |  |
| **Childhood cancer type** | **leukaemia** | 4093 (24.6%) | 7064 (42.4%) | 5489 (33.0%) | 16646 |
|  | **Hodgin lymphoma** | 558 (9.2%) | 2329 (38.5%) | 3159 (52.3%) | 6046 |
|  | **non-Hodgkin lymphoma** | 1262 (31.0%) | 998 (24.5%) | 1818 (44.6%) | 4078 |
|  | **CNS tumour^b^** | 2796 (19.2%) | 4328 (29.7%) | 7468 (51.2%) | 14592 |
|  | **neuroblastoma** | 1326 (41.7%) | 771 (24.4%) | 1081 (34.0%) | 3178 |
|  | **retinoblastoma** | 642 (24.8%) | 731 (28.2%) | 1217 (47.0%) | 2590 |
|  | **Wilms tumour** | 1219 (25.5%) | 1859 (38.9%) | 1705 (35.7%) | 4783 |
|  | **bone sarcoma** | 948 (29.9%) | 824 (26.0%) | 1401 (44.2%) | 3173 |
|  | **soft-tissue sarcoma** | 1160 (25.6%) | 1328 (29.3%) | 2043 (45.1%) | 4531 |
|  | **other** | 177 (20.8%) | 170 (30.4%) | 948 (46.5%) | 9843 |
| Total | | 16051 (23.1%) | 21126 (30.4%) | 32283 (46.5%) | 69460 |
| Abbreviations: CNS; Central nervous system  ^a^Information on presence or absence of radiotherapy treatment for treatment of the childhood cancer was available for 37,177 of 69460 (53.5%) survivors. ^b^Note that for all Nordic countries (Iceland, Norway, Sweden, Denmark, Finland) radiotherapy treatment information was completely missing; excluding the Nordic countries the amount of missing treatment was 10.5% overall. | | | | | |

| **eTable 3.** Age-specific number of individuals at risk of developing a glioma or meningioma SPN | | | | | | |
| --- | --- | --- | --- | --- | --- | --- |
|  | | **Attained Age (yrs)** | | | | |
|  |  | **20** | **30** | **40** | **50** | **60** |
| **CNS**  **SPN type** | **Glioma** | 44,092 | 29,738 | 16,069 | 6,296 | 1,825 |
|  | **Meningioma** | 44,083 | 29,626 | 15,895 | 6,223 | 1,806 |
| Abbreviations: CNS; Central nervous system, SPN; Subsequent Primary Neoplasm | | | | | | |


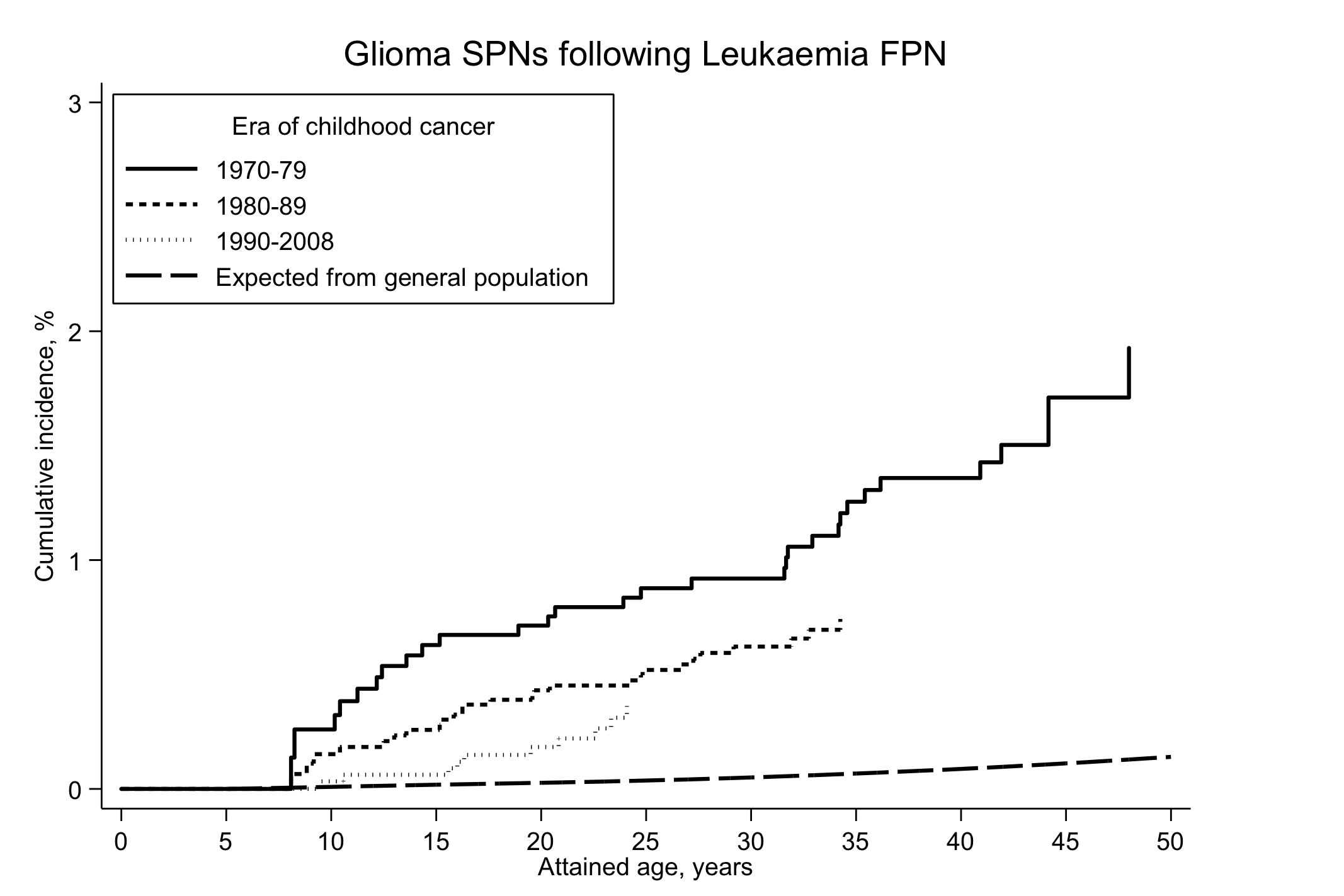


50yrs:

1.9%

35yrs:

0.7%

25yrs:

0.4%

eAppendix figure 1: Cumulative incidence of gliomas following childhood leukaemia by era of diagnosis

Cumulative Incidence for survivors diagnosed in 1970-79 at 50 years: 1.9% (95% CI: 1.2%, 2.8%)

Cumulative Incidence for survivors diagnosed in 1980-89 at 35 years: 0.7% (95% CI: 0.5%, 1.1%)

Cumulative Incidence for survivors diagnosed in 1990-2008 at 25 years: 0.4% (95% CI: 0.2%, 0.7%)


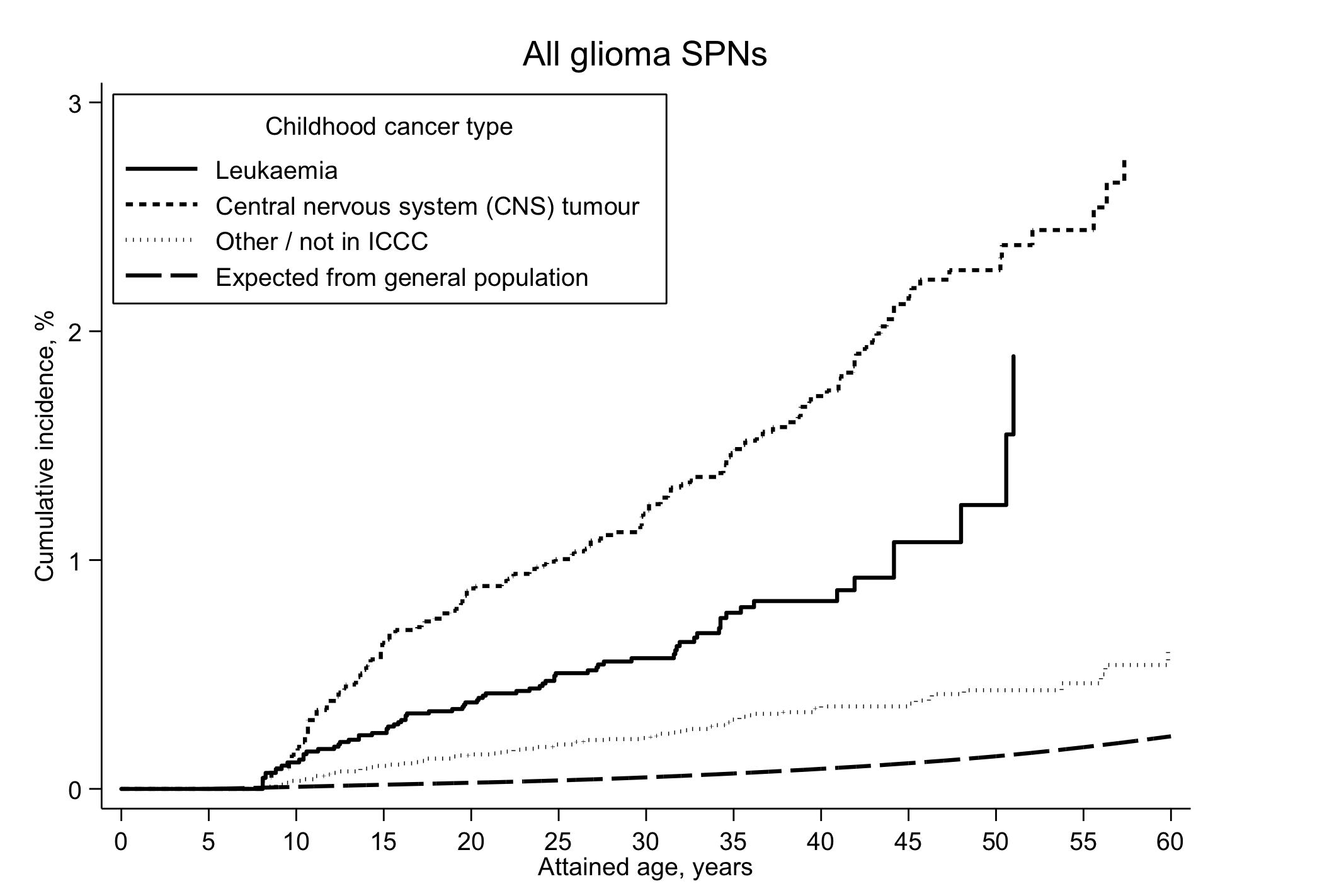


eAppendix figure 2: Cumulative incidence of gliomas by childhood cancer type


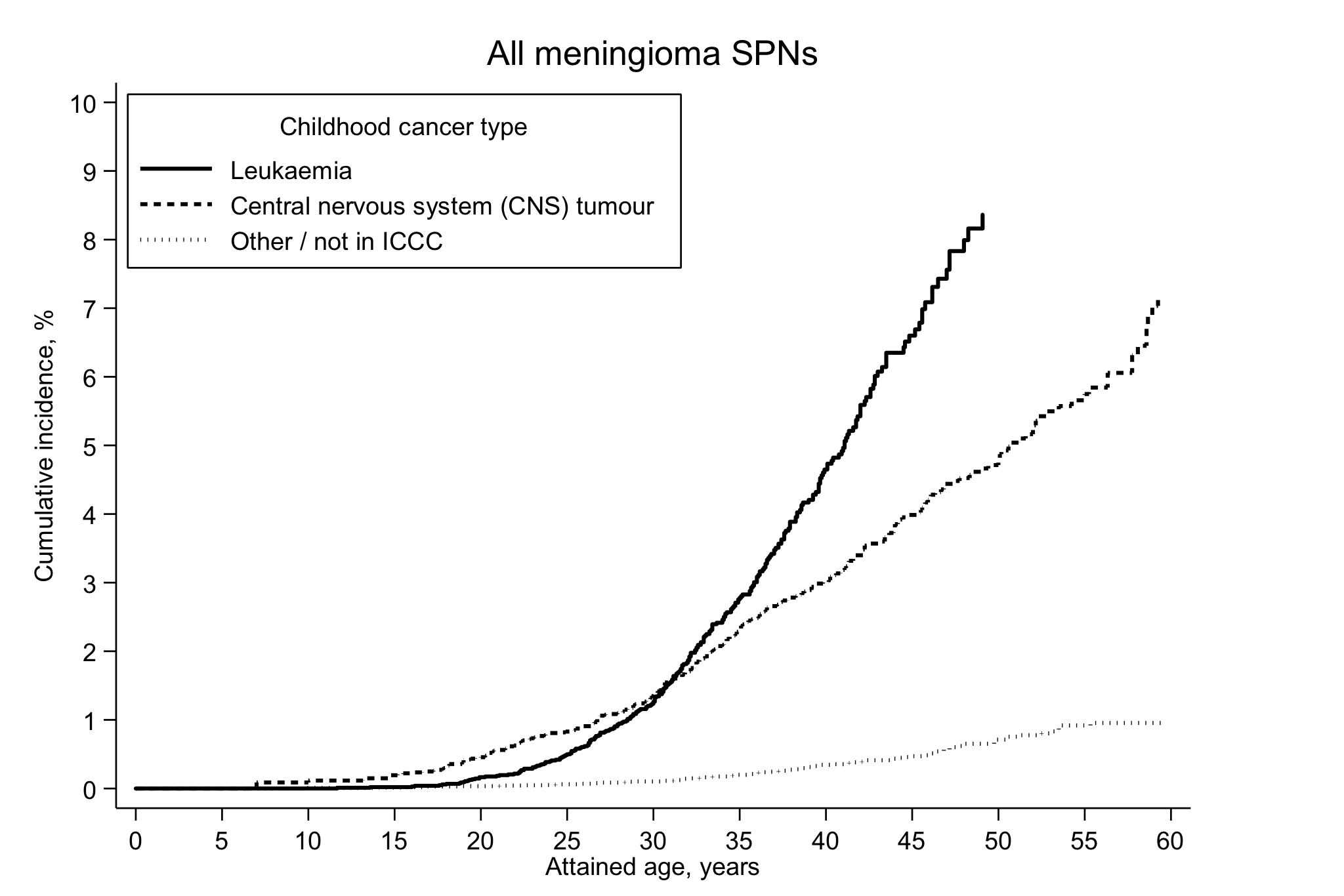


eAppendix figure 3: Cumulative incidence of meningiomas by childhood cancer type
